# Induced pluripotent stem cells as platforms for engineering NK cell immunotherapies

**DOI:** 10.3389/fcell.2026.1810206

**Published:** 2026-05-01

**Authors:** Anna Jezierski, Jez Huang, Lea Desbiens, Basma Benabdallah, Scott McComb, Christian Beausejour

**Affiliations:** 1 Human Health Therapeutics Research Center, Translational Biosciences, National Research Council of Canada, Ottawa, ON, Canada; 2 Ottawa Institute of Systems Biology, University of Ottawa, Ottawa, ON, Canada; 3 Department of Biochemistry, Microbiology and Immunology, Faculty of Medicine, University of Ottawa, Ottawa, ON, Canada; 4 Centre de Recherche du CHU Ste-Justine, Montréal, QC, Canada; 5 Département de Pharmacologie et Physiologie, Université de Montréal, Montréal, QC, Canada

**Keywords:** chimeric antigen receptor (CAR), genome engineering, immunotherapy, induced pluripotent stem (iPS) cells, natural killer cells

## Abstract

Human induced pluripotent stem cells (iPSCs) are transforming adoptive cell therapy by combining unlimited self-renewal, broad differentiation potential, and high amenability to genome engineering. These attributes make iPSCs a versatile source for the development of standardized immune effector cells at industrial scale, enabling a shift from patient- or donor-restricted cell products toward true off-the-shelf immunotherapies that can be improved through iterative genome engineering. iPSC-derived natural killer (iNK) cells are the most clinically advanced and exemplify the platform’s advantages over conventional autologous or donor-sourced approaches. Unlike autologous therapies, which require labor-intensive and expensive personalized clinical-grade manufacturing, and are constrained by variable quality and genetic intractability of donor products, iPSC technology supports the creation of renewable, clonally defined master cell banks as uniform starting material for NK-cell therapy products. Advances in CRISPR/Cas-based editing now permit multiplex introduction of functional traits, enhanced cytokine signaling, antibody-dependent cytotoxicity, checkpoint resistance, optimized trafficking, safety switches, and increasing signal complexity, directly at the pluripotent or progenitor stages; ultimately allowing for fully-programmable iNK cells with customizable potency and persistence. Early clinical studies of iNK products validate the feasibility, safety, and therapeutic potential of this approach, but also underscore the need for continued refinement of differentiation protocols, manufacturing pipelines, and regulatory standards to ensure efficacy, genomic stability, phenotypic maturity, and long-term safety. This review outlines current breakthroughs and future directions of iNK cell therapies, emphasizing how programmable iPSC chassis platforms are enabling modular and off-the-shelf targeted immunotherapies.

## Pluripotent stem cells at the forefront of next-generation immunotherapy

Pluripotent stem cells (PSCs), including embryonic stem cells (ESCs) and induced pluripotent stem cells (iPSCs), have transformed our understanding of human development, and disease modeling while redefining the landscape of regenerative medicine and immune-based therapeutics. With their intrinsic ability to self-renew indefinitely and differentiate into any somatic lineage, PSCs have emerged as a versatile platform for the development of engineered cell-based therapeutics.

Human iPSCs represent a paradigm shift in the design of next-generation immunotherapy, offering high amenability to genome engineering and the capacity to generate large, standardized master cell banks for off-the-shelf therapeutic applications. As such, iPSC-derived Natural Killer (iNK) cell therapies offer a different manufacturing paradigm compared with conventional autologous and allogeneic donor-derived products. Autologous NK therapies, while fully personalized, require scale-out, clinical-grade manufacturing for each individual, which is labor-intensive, time-consuming, expensive, and typically produces only a limited number of doses per patient. Donor-derived allogeneic NK cells, typically sourced from peripheral blood mononuclear cells (PBMCs), offer the advantages of off-the-shelf, donor-to-batch availability, centralized scale-up manufacturing, and the potential to generate many doses from a single donor. They also often display a more mature NK cell phenotype and can be expanded and banked for distribution. However, their utility is limited by donor-dependent cellular variability, batch-to-batch heterogeneity, and a supply chain constrained by donor availability, recruitment and screening. Additionally, these cells often display limited *in vivo* persistence without exogenous cytokine support and are inherently difficult to genetically modify. Most critically, regardless of whether T, NK, or other cell types are employed in allogeneic or autologous format, the finite capacity of peripheral cells to self-renew limits the number of iterative or multiplex genomic edits that can be applied to differentiated cell therapies, making it difficult (if not impossible) to build directly upon the success of any one therapeutic approach through iterative engineering.

**FIGURE 1:**
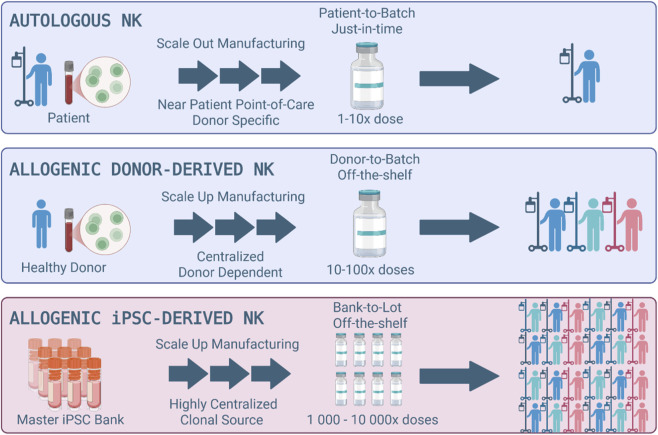
Comparison of biomanufacturing approaches for autologous, allogeneic donor-derived, and allogeneic iPSC-derived NK therapies. Autologous NK therapies are patient-specific, manufactured on a just-in-time basis, and yield a limited number of doses per patient. Allogeneic donor-derived NK therapies enable donor-to-batch off-the-shelf use and modest scalability, producing tens to hundreds of doses per donor. In contrast, iPSC-derived NK cell therapies originate from an engineered iPSC master cell bank enabling bank-to-lot industrial-scale manufacturing of tens of thousands of standardized, consistent and homogenous off-the-shelf doses. This approach offers unmatched scalability, consistency, and accessibility for broad clinical application. Figure created using BioRender.com and adapted from (Evotec, 2025).

In contrast, iPSC platforms enable the generation of renewable, clonally defined master cell banks that serve as uniform, well-characterized starting material at industrial scale. Unlike autologous or donor-derived allogeneic sources, iPSCs support true off-the-shelf, bank-to-lot scale up manufacturing, decoupling production from donor- or patient-specific variability, while permitting stringent quality control, long-term cryopreservation, and iterative genome engineering at the pluripotent or progenitor stages. Among iPSC-derived immune lineages under clinical development, iPSC-derived NK (iNK) cells are the most advanced, exemplifying the capacity of the platform to generate thousands of standardized, off-the-shelf doses from a single clonally derived master bank with uniform phenotype, potency, and effector functionality significantly shortening time-to-therapy. Comparative manufacturing paradigms for autologous and allogeneic donor- and iPSC-derived NK cells are illustrated in Figure 1, with a summary of their relative advantages and limitations provided in Table 1. Notably, these differences do not primarily stem from a need for near-patient or point-of-care manufacturing, but rather from the stringent process controls and regulatory requirements inherent to GMP-compliant production at scale. As genome editing technologies advance, particularly CRISPR/Cas-based methods, iNK cells are becoming highly programmable, enabling precise tuning of persistence, trafficking, and effector function to address diverse clinical indications.

**TABLE 1:** Comparative biomanufacturing, clinical, and scalability advantages and limitations of autologous, allogeneic donor-derived, and iPSC-derived NK cell therapies.

NK therapy type	Advantages	Disadvantages
Autologous	-Patient-to-Batch personalized dose -Well-characterized donor history -Ability to leverage patient-specific tumor antigens -No donor recruitment or screening required	- High fixed costs for scale-out manufacturing timelines (often point of care) - Small yield per batch (limited scalability) - Limited opportunity for genetic engineering - Compromised cell health and quality of donor cells due to disease/treatments
Allogeneic Donor-Derived	- Donor-to-Batch Off-the-shelf availability - Donor screening/selection -More mature NK phenotype - Scale-up centralized biomanufacturing -Banked and distributable product (multiple doses per donor)	- Challenging to genetically manipulate - Batch-to-batch variability in quality and heterogeneity of donor cells - Donor-dependent supply chain; recruitment/screening - Limited scalability (batch per donor) -Short persistence without exogenous cytokine support
Allogeneic iPSC-Derived	-Bank-to-Lot Off-the-Shelf availability - Renewable master iPSC cell bank -Highly amenable to genetic engineering -Homogeneous, consistent and standardized product - Scale-up centralized biomanufacturing -Unlimited industrial scale expansion and supply chain	-Limited clinical track record (emerging field) -High upfront development costs - Potentially immature NK cell phenotype - Higher Chemistry, Manufacturing, Control (CMC) burden -Genomic instability and safety risk of residual undifferentiated iPSCs - Lack of global regulatory alignment on requirements

## Advancing iPSC immunotherapies through genome editing

A key inflection point in the evolution of iPSC-derived immunotherapies has been the integration of genome engineering technologies, most notably, CRISPR/Cas systems (reviewed in Fang et al., 2025). Unlike primary or donor-derived NK cells, which are notoriously difficult to genetically manipulate due to low susceptibility to viral and non-viral gene transfer, donor-to-donor variability in editing efficiency, and hence limited opportunity for iterative engineering (Naeimi Kararoudi et al., 2020), iPSCs readily tolerate multiplex genome edits and can be clonally selected and expanded to generate homogeneous, engineered cell products suitable for standardized manufacturing. The simplicity, programmability, and multiplexing capabilities of CRISPR allow the introduction or deletion of specific genetic elements with high fidelity, thereby facilitating the development of custom-engineered cell products with improved therapeutic attributes such as safety, efficacy, and allogeneic compatibility (Kumar et al., 2025; Lei et al., 2024).

**FIGURE 2:**
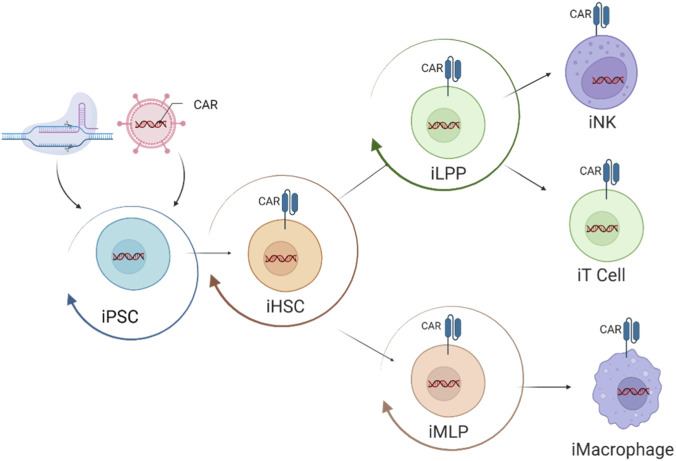
Schematic of iPSC-derived immune cell CAR engineering and differentiation. Human iPSCs can be genetically modified at the pluripotent stage to incorporate functional attributes such as CARs. Engineered iPSCs are differentiated into hematopoietic stem cells (iHSCs), which give rise to intermediate progenitors, including lymphoid progenitors (iLPP) and myeloid lineage progenitors (iMLP). These progenitors can subsequently be differentiated into multiple immune effector lineages, including iNK cells, iT cells, and iMacrophages, all carrying the CAR and any additional engineered genetic alterations. Figure created using BioRender.com.

Autologous, chimeric antigen receptor T cell (CAR-T) therapies have now become standard therapy for relapsed B cell and plasma cell malignancies (Zugasti et al., 2025). This clinical success has helped drive the broader adoption of CAR engineering across other immune cell platforms and CARs are now routinely integrated into iPSC-derived immune cells, enabling the generation of cell therapies with customizable antigen specificity and enhanced antitumor activity (Alidadi et al., 2024). Importantly, this editing can be performed at the pluripotent or early progenitor stage, enabling the introduction of CARs before lineage commitment (Figure 2). By doing so, a single engineered iPSC line can be directed toward multiple immune effector lineages such as iT cells, iNK cells or iMacrophages, each carrying identical CAR configurations or therapeutic attributes tailored for specific effector functions. This master iPSC bank approach allows for the scalable and reproducible production of diverse CAR-modified immune cell types from a single source, streamlining manufacturing and quality control. Moreover, it opens the door to next-generation combination immunotherapies, where dual or synergistic CAR-T, CAR-NK and CAR-macrophage cell therapies, derived from the same clonal origin, can be deployed for a multi-pronged therapeutic strategy, targeting the heterogeneous tumor landscapes through complementary mechanisms of action. The modular nature of iPSC and CRISPR/Cas systems also supports rapid iterative design and optimization, driving a robust pipeline of living drugs capable of addressing complex diseases with improved efficacy and safety. This broad framework sets the stage for more detailed exploration of specific iPSC-derived immunotherapies which are discussed in the following sections.

## Advances in iPSC-derived CAR NK cell engineering

The development of iNK cell therapies has rapidly progressed from proof-of-concept to advanced engineered cell therapy candidates with enhanced therapeutic efficacy. Initially constrained by limited persistence, expansion, and cytotoxicity, particularly in the context of solid tumors, iNK platforms are now being reshaped through innovative genetic and molecular strategies (Kumar et al., 2025; Xiong and Zheng, 2024). A central focus of these engineering efforts has been on optimizing cytokine signaling, given its critical role in sustaining NK cell survival and function. Interleukin-15 (IL-15) is particularly indispensable, driving NK cell maturation, persistence, and antitumor activity (Mehta et al., 2022). However, due to IL-15’s inherently short half-life, repeated supplementation is often required during *in vitro* culture to maintain NK cell activity. To overcome this limitation, engineered IL-15 constructs, such as secreted IL-15 or membrane-bound IL-15/IL-15Rα fusion proteins, have been designed to provide sustained cytokine signaling, thereby supporting continuous iNK proliferation, survival, and antitumor activity without the need for exogenous supplementation (Woan et al., 2021). Complementing this strategy, knockout of cytokine-inducible SH2-containing protein (CISH), a feedback inhibitor of IL-15 signaling, further enhances IL-15 mediated JAK-STAT activation, amplifying the functional persistence and cytotoxic potential of iNK cells (Zhu et al., 2020a).

In addition to cytokine-based enhancements, strategies to improve antibody-dependent cellular cytotoxicity (ADCC) have also advanced iNK cell functionality. CD16a, the principal Fc receptor mediating ADCC in NK cells, typically has low affinity for IgG and is prone to shedding upon activation due to cleavage by the metalloprotease ADAM17. To circumvent these limitations, one strategy involved fusing CD64, an Fcγ receptor with the highest affinity for IgG, with CD16a, while removing the ADAM17 cleavage site (Snyder et al., 2018). This CD64/16a fusion protein conferred improved antibody binding, reduced downregulation upon activation, and enhanced ADCC, cytokine release, and tumor cell killing (Dixon et al., 2020; Dixon et al., 2024). Another approach utilized a high-affinity, non-cleavable mutant of CD16a (hnCD16), which when introduced into iNK cells, conferred mature functionality and robust ADCC against diverse tumor types. In xenograft models of B-cell lymphoma, treatment with hnCD16-expressing iNK cells, in combination with anti-CD20 monoclonal antibodies, led to marked tumor regression and prolonged survival (Zhu et al., 2020b). Building on this, a further refinement involved fusing the extracellular domain of hnCD16 with NK-specific activating intracellular signaling domains, resulting in enhanced cytotoxic responses *in vitro* and *in vivo*, including in human B-cell lymphoma xenografts (Meng et al., 2023).

Several other strategies have been developed to enhance the cytotoxic effects of iNK cells. For example, knocking out CD38 and introducing CD16 resulted in improved anti-tumor activity, particularly in myeloma (Stikvoort et al., 2021). CD38 is a multifunctional ectoenzyme that metabolizes NAD^+^ to produce immunosuppressive adenosine. Hence, triple-engineered iNK cells expressing hnCD16a, a membrane-bound IL-15/IL-15Rα fusion protein, and a CD38 knockout exhibit enhanced antitumor activity against leukemia and multiple myeloma (Woan et al., 2021). Similarly, triple-engineered CD70-targeted iPSC-CAR-NK (70CAR-iNK) cells incorporating CD70 knockout prevents fratricide by eliminating self-recognition of the CAR target together with hnCD16, and IL-15/IL-15Rα fusion protein showed robust cytotoxicity against diverse tumors, eliminated alloreactive T cells, and demonstrated enhanced *in vivo* persistence (Wang et al., 2025). Lastly, engineered iNK cells expressing CCL19, CCR2B, hnCD16, IL-15, NKG2D, and DAP10 resulted in increased antitumor activity. In this design, CCR2B enhanced tumor infiltration by improving chemotactic responsiveness, while CCL19 promoted recruitment of dendritic cells to the tumor microenvironment. The combined expression of hnCD16, IL-15, NKG2D, and DAP10 synergistically augmented NK cell activation, persistence, and cytotoxicity (Fukutani et al., 2025).

NKG2A is an inhibitory receptor on NK cells that recognizes HLA-E, suppressing their cytotoxic activity. Hence, knocking out the *NKG2A* gene in iNK cells was shown to enhance their ability to eliminate tumor cells, particularly those with high HLA-E expression (Kamiya et al., 2019; Qin et al., 2024). NKG2C has also emerged as an attractive immunotherapeutic target, as this activating NK cell receptor delivers potent stimulatory signals and is associated with memory-like NK cell responses. Recently, a tri-specific NK cell engager (NKCE) incorporating NKG2C, IL-15, and CD33 was engineered to selectively activate NKG2C-expressing iNK cells at the tumor site. This construct enhanced NK cell functional activity, including elevated degranulation and IFN-γ secretion, and mediated robust cytotoxic effects against CD33-expressing tumor targets, including primary acute myeloid leukemia (AML) (Chiu et al., 2021).

Transforming growth factor beta (TGF-β) is highly expressed in the hepatocellular carcinoma (HCC) tumor microenvironment and is known to inhibit immune cell activity. To overcome this immunosuppressive microenvironment, iNK cells were engineered to either knockout TGF-β receptor 2 (TGFBR2-KO) or express a dominant-negative form of TGFBR2 (TGFNR2-DN), together with CARs targeting glypican-3 (GPC3) or alpha-fetoprotein (AFP) (Thangaraj et al., 2024). Both TGFBR2-KO and TGFNR2-DN iNKs were resistant to TGF-β-mediated immunosuppression and exhibited enhanced cytotoxicity and anti-tumor activity. However, engineering iNK cells with anti-HCC CARs alone was not sufficient to produce effective anti-tumor activity unless TGF-β signaling was simultaneously blocked. These findings position TGF-β resistance as a critical determinant of NK cell efficacy in HCC, with potential relevance to other TGF-β-abundant solid tumors. In addition to soluble suppressive factors such as TGF-β, tumor cells also exploit glycan-mediated immune evasion mechanisms through surface sialylation, which engages inhibitory receptors such as SIGLEC7 on NK cells. SIGLEC7-KO iNK cells showed significantly enhanced degranulation (CD107a) and increased IFN-γ and TNF-α production, with functional activity comparable to that of TGFBR2-KO iNK cells (Thangaraj et al., 2025). Together, these findings suggest that, in addition to overcoming glyco-immune checkpoint inhibition, SIGLEC7 deletion may confer benefits similar to disruption of TGF-β signaling, highlighting its potential as an effective strategy to enhance iNK cell function in suppressive tumor microenvironments.

**FIGURE 3:**
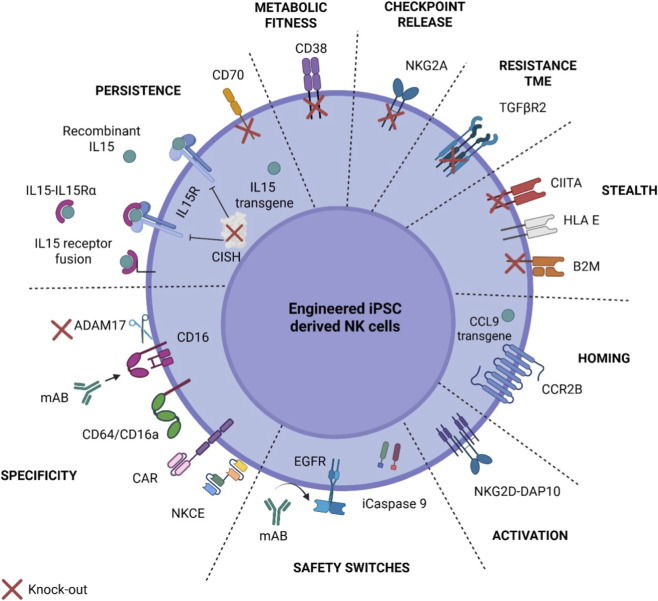
Engineering strategies in iNK cells. Schematic overview of protein and genome engineering approaches used to enhance the therapeutic potential of engineered iNK cells. Modifications are grouped by functional category: Persistence (IL-15 transgene, IL-15 receptor fusion (IL15-IL15Rα), recombinant IL-15, CISH knockout, CD70 expression, ADAM17 knock out), Metabolic Fitness (CD38 knockout), Checkpoint Release (NKG2A knockout), Resistance to Tumor Microenvironment (TME) (TGFβR2 knockout), Immune Stealth (B2M and CIITA knockouts, HLA-E knock in), Homing (CCR2B, CCL9 knock-in), Activation (NKG2D-DAP10, hnCD16), Specificity (CARs, CD16/CD64a engagement, Fc-engagers, monoclonal antibody cooperation, NKCE), and Safety Switches (iCaspase 9, truncated EGFR). The figure highlights how combinatorial engineering approaches target multiple aspects of NK cell biology to improve efficacy, persistence, and safety in adoptive cell therapy applications. Figure created using BioRender.com and adapted from (Fang et al., 2024).

Lastly, using a synNotch-based strategy, iNK cells have been engineered to conditionally express therapeutic payloads, such as anti-CD73 antibodies, upon engagement with tumor antigens like CD155. In this context, synNotch functions as a programmable logic gate that senses CD155 and triggers a transcriptional cascade to block CD73-mediated adenosine production, strategically flipping immunosuppressive cues into immunostimulatory action within glioblastoma (Lupo et al., 2024). Collectively, these strategies highlight the intrinsic compatibility of iPSC-derived platforms with high-throughput, multiplex genome editing, thereby permitting the rational design and engineering of therapeutic attributes within a single, uniform NK cell product. A summary of these engineered therapeutic attributes in iNK cells is depicted in Figure 3 and further reviewed in (Kumar et al., 2025).

## Mapping functional regulators for next-generation iNK cells

Genome engineering strategies to enhance iNK cell function have made significant progress in improving their therapeutic outcomes (Figure 3); however, the full spectrum of genetic targets that could further improve persistence, cytotoxicity, and tumor specificity remains largely unexplored. In particular, targets to overcome tumor- and microenvironment-mediated immunosuppression remain to be fully defined. Genome-wide CRISPR screens provide a powerful platform to uncover both intuitive and non-obvious regulators of NK function, particularly when performed under tumor-like stress conditions, including hypoxia, TGFβ exposure, and nutrient deprivation. Insights from multiple screens in primary NK cells have identified key checkpoints controlling resistance to immunosuppressive pressures; for example, loss of genes such as *MED12*, *ARIH2*, *CCNC,* and *UBE2F* significantly enhanced antitumor activity (Biederstädt et al., 2025; Nikolic et al., 2025), suggesting that analogous modifications could also improve iNK cell function. Integration of genome-wide perturbations with multi-omic profiling such as single-cell transcriptomics, proteomics, and epigenomics enables the mapping of functional programs, identification of context-specific vulnerabilities, and discovery of synergistic or combinatorial targets. Iterative testing of these target modifications in iNK cells provides a scalable and clonally defined platform to systemically engineer and validate novel therapeutic modalities. Collectively, these strategies can provide a roadmap for rationally designing next-generation iNK cell therapies with optimized potency, specificity, and resilience against diverse, treatment-refractory cancers.

## Clinical translation of CAR-iNK therapies: linking design to outcome

**FIGURE 4:**
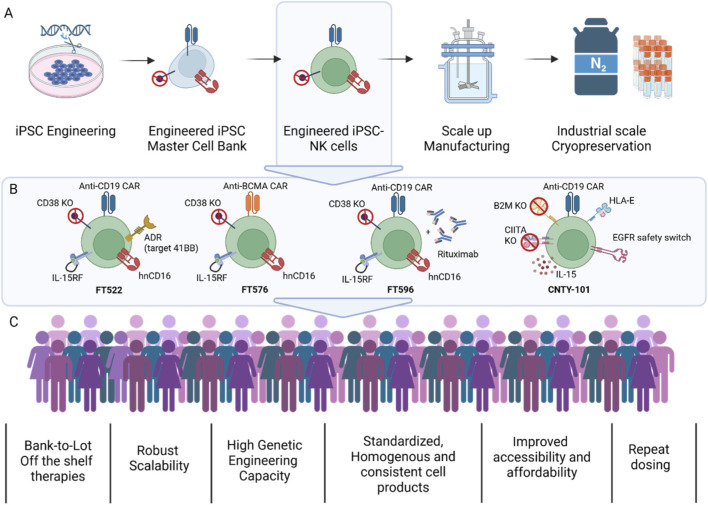
Workflow for the generation and clinical application of iNK cells. **(A)** Workflow for generating iPSC-derived, genetically engineered master cell banks incorporating anti-tumor modalities (e.g., cytokine support, safety switches, persistence and efficacy etc). These master banks serve as a renewable source for producing engineered therapeutic iNK cells, which can be scaled, under controlled conditions, to yield defined clinical-grade cell products. **(B)** Schematic illustrating the engineered therapeutic attributes of anti-CD19 and BCMA-iNK candidates (FT522, FT576, FT596 and CNTY-101) in clinical trials. **(C)** Key advantages of iNK therapies in overcoming limitations of autologous CAR-NK approaches enabling the generation of consistent, scalable, and off-the-shelf cell-based treatments. Figure created using BioRender.com.

The first wave of clinical trials for iPSC-derived CAR-iNK cell therapies is beginning to validate the promise of these engineered, off-the-shelf platforms, demonstrating how precise molecular designs translate into distinct clinical phenotypes (Figure 4a). The clinical pipeline illustrates how specific genomic engineering strategies directly shape the therapeutic profile of each iPSC-derived product, with precise combinations of gene knockouts, knock-ins, and transgene insertions tailored to enhance efficacy, safety, and persistence (Kirkeby et al., 2025) (Figures 4b,c). For examples, Fate Therapeutics’ FT522 incorporates a CD38 knockout, hnCD16, an IL-15/IL-15Rα fusion, and a CD19 CAR, a design also echoed in FT596, which showed durable responses across multiple lymphoma subtypes, even in patients relapsed after CAR-T therapy (Bachanova et al., 2021). Similarly, FT516, though lacking a CAR, leverages hnCD16 and IL-15 signaling to achieve a 72% objective response rate at higher doses, including complete responses in CAR-T refractory patients (Patel et al., 2021). For multiple myeloma, FT576 combines a BCMA CAR with CD38 KO, hnCD16, and IL-15, demonstrating potent protective effects in preclinical models (Dhakal et al., 2022; Goodridge et al., 2021). Century Therapeutics’ CNTY-101, CAR-iNK product, features a CD19 CAR, secreted IL-15, a truncated epidermal growth factor receptor (EGFR) safety switch, and genome edits including B2M and CIITA knockouts and HLA-E knock-in (Patel et al., 2024; Ramachandran et al., 2023). The inclusion of a modified version of EGFR incorporated into the CAR construct serves as a target for a clinically available antibody, cetuximab, allowing for the targeted elimination of CAR-iNK cells if severe side effects like cytokine release syndrome (CRS) occur. This product has shown tumor shrinkage and durable responses, with tumor profiling indicating enhanced activation of the adaptive immune response (Patel et al., 2024). In addition to these clinical candidates, the commercial landscape of companies developing iNK cell products continues to grow (reviewed in Kumar et al., 2025; Romanini et al., 2025) highlight how rational multiplex genomic design underpins functional outcomes in next-generation, off-the-shelf cellular immunotherapies.

These clinical trials of CAR-iNK therapies have shown encouraging safety profiles, with minimal severe toxicities reported to date with patient doses from 30 million to 1 billion cells (summarized in Table 2) (Kirkeby et al., 2025). This aligns with the intrinsic biology of NK cells, which lack the capacity to induce graft-versus-host disease in an allogeneic setting and are associated with a lower risk of cytokine release syndrome (CRS) and immune effector cell-associated neurotoxicity syndrome (ICANS) (Wang W. et al., 2024). However, it remains to be determined if CAR-NK, especially CAR-iNK, will match CAR-T in their cytotoxic potential (Egli et al., 2024). Clinical iNK cell products such as FT522, FT516, FT576 and CNTY-101, as well as iT cell product FT819 demonstrated low rates of CRS (mostly grade 1–2) and no instances of neurotoxicity, graft-versus-host disease (GvHD), or dose-limiting toxicities (Bachanova et al., 2021; Dhakal et al., 2022; Mehta et al., 2022; Patel et al., 2021; Patel et al., 2024). FT596, tested in a larger patient cohort, showed low-grade CRS in 12% of cases without neurotoxic effects (Bachanova et al., 2021; Ghobadi et al., 2025). Transient cytopenias were observed, particularly with FT522 and FT516, but were manageable (Bachanova et al., 2024). Notably, CNTY-101 did not trigger anti-drug antibodies and was well tolerated across dose levels. Collectively, these outcomes highlight both the promising early activity and benign safety profile of iNK cell products and the platform’s capacity for multiplex genome editing. (Figure 3). Nevertheless, these results are based on small, early-phase cohorts and limited follow-up, underscoring the need for larger, randomized studies with extended monitoring to fully establish durability, comparative efficacy, and long-term safety.

**TABLE 2:** Overview of clinical FT522, FT576, FT596, and CNTY-101 iPSC-iNK clinical candidates: Engineering Edits, Development Phase, and Emerging Clinical Outcomes.

Product	Company	Key edits/Engineering features	Clinical phase/Status	Reported clinical/Early outcomes
FT522	Fate Therapeutics	iNK CD19-CAR Alloimmune Defense Receptor (ADR) – 41BB-CAR; CD19 targeting; hnCD16 IL-15/IL-15 fusion CD38-null	Phase 1 in relapsed/refractory B-cell lymphoma (NCT05950334); also, being trialed in autoimmune disease indications 20 patients	*Safety*: No DLTs, no serious CRS, ICANS, or GvHD *Efficacy*: Indolent lymphoma 3/3 CRs; Mantle cell lymphoma PR; DLBCL – no response at low dose, 1CR, 1PR at high dose (Bachanova et al., 2024)
FT576	Fate Therapeutics	iNK BCMA-CAR; high-affinity 158V; hnCD16 IL15/IL15R fusion CD38-null	Phase 1 (called FT576-101) in relapsed/refractory multiple myeloma (MM) (NCT05182073) ‒ monotherapy and in combination with daratumumab 31 patients	*Safety*: No CRS, ICANS, or GvHD observed; some Grade ≥3 AEs (diarrhea, neutropenia, anemia) but these resolved *Efficacy*: 1/9 pts VGPR, 1/9 MR, 1/9 PR 4/9 stable disease (SD). (Dhakal et al., 2022; Goodridge et al., 2021)
FT596	Fate Therapeutics	iNK; CD19-CAR hnCD16 IL15/IL15R fusion	Phase 1 in relapsed/refractory B-cell lymphoma and chronic lymphocytic leukemia (NCT04245722) ‒ with standard conditioning + rituximab or obinutuzumab 98 patients	*Safety*: Low-grade CRS; no ICANS or GvHD; Grade ≥3 higher infections reported in 20% of patients *Efficacy*: Follicular lymphoma: 11/13 CR, Median duration of response ∼16.9 months DLBCL 5% 1/21 CR, Median duration not yet reached (9.1 months). (Ghobadi et al., 2025)
CNTY-101/ELiPSE-1	Century Therapeutics	iNK; CD19-CAR; -iNK product; IL-15; precision gene edits for “Allo-Evasion” (B2M and CIITA knock outs, HLA-E knock in)	Phase 1 (“ELiPSE-1”) trial for relapsed/refractory CD19^+^ B-cell malignancies. (Trial discontinued) (NCT05336409) 28 patients	*Safety:* All doses of CNTY 101 showed SD. No DLTs, GvHD or ICANS were observed. 2 patients had CRS. *Efficacy:* Across higher-dose cohorts (1 × 10^9^ cells), overall response rate (ORR) was 67% and complete response rate (CRR) ∼33%. Patient achieved a complete response (CR) sustained for 5 months (Patel et al., 2024; Ramachandran et al., 2023)

Abbreviations: iNK, iPSC-derived Natural Killer Cells; CAR, Chimeric Antigen Receptor; ADR, Alloimmune Defense Receptor; KO, Knock-out; DLT, Dose-limiting toxicity; CRS, Cytokine Release Syndrome; ICANS, Immune effector cell-associated neurotoxicity syndrome; GvHD, Graft-versus-host disease; CR, Complete response; PR, Partial response; DLBCL, Diffuse Large B-cell Lymphoma; BCMA, B-Cell maturation antigen; MM, Multiple Myeloma; AE, Adverse event; VGPR, Very good partial response; MR, Minimal response; SD, Stable disease; CLL, chronic lymphocytic leukemia; SLE, Systemic Lupus Erythematosus; ORR, Objective response rate; CRR, complete response rate; DLT, Dose-limiting toxicity; CRS, Cytokine release syndrome; ICANS, Immune effector cell-associated neurotoxicity syndrome; GvHD, Graft-versus-host disease; R/R, Relapsed/refractory; NHL, Non-Hodgkin’s lymphoma; RP2D, Recommended Phase-2 dose.

## Bridging the gap: navigating barriers in iPSC-derived immune cell therapies

### Overcoming engineering hurdles in iPSC-Based immunotherapies

Despite remarkable progress in the development of iNK cell therapies, several challenges remain to be overcome to realize their full clinical potential (reviewed in Romanini et al., 2025). One of the primary concerns is maintaining genomic integrity throughout the reprogramming and complex genetic engineering required to equip these cells with clinically relevant therapeutic properties (Fang et al., 2024; Madrid et al., 2024; Steichen et al., 2019). Multiple rounds of genome editing, particularly when involving insertion of large transgenes, multiplexed knockouts, or synthetic gene circuits, can introduce unintended mutations and off-target effects, chromosomal rearrangements, or epigenetic instability potential leading to increased risk of malignant transformation, impaired cell function, or reduced therapeutic safety and efficacy (Dashtban et al., 2020; Doss and Sachinidis, 2019). To maintain genetic and clonal integrity, thorough genomic monitoring should be conducted across all stages of reprogramming, gene editing, and cell expansion. For example, CRISPR-Cas9 editing the TRAC locus in T cells to enable allogeneic application has been linked to large-scale deletions on chromosome 14 in a subset of cells (Tsuchida et al., 2023). To address these risks, non-cutting CRISPR systems such as base and prime editing are being actively explored, along with complementary approaches like CRISPR interference (CRISPRi), CRISPR activation (CRISPRa), epigenome editing, and RNA-targeting platforms like Cas13, which enable precise and reversible modulation of gene expression without permanent genomic alterations (Chen and Liu, 2023; Lei et al., 2024). Recent work has demonstrated, for the first time, that base editors can be efficiently applied in primary human NK cells to introduce both loss- and gain-of-function mutations, resulting in enhanced cytotoxic activity (Wang M. et al., 2024). Moreover, when multiplex base editing was combined with non-viral transposon-mediated integration to generate IL-15 CD19 CAR-NK cells, the resulting product exhibited superior antitumor efficacy *in vitro* and *in vivo* within a highly suppressive Burkitt’s lymphoma model (Wang M. et al., 2024). Together, these advances underscore the transformative potential of multiplex base editing, particularly when integrated with non-viral delivery systems, to create safer, more potent, and customizable NK cell therapies. This proof of concept establishes a strong foundation for extending such strategies to iNK cells, enabling precise, scalable, and multiplex engineering for next-generation immunotherapies.

Off-target effects remain a major challenge in the genetic engineering of iPSC-derived immunotherapies. To mitigate this, high-fidelity CRISPR nucleases such as SpCas9-HF1, eSpCas9, and HypaCas9 have been developed to improve on-target precision without compromising editing efficiency (Chen et al., 2017; Fang et al., 2025). In parallel, targeting transgenes to genomic “safe harbor” loci, such as AAVS1, CCR5, or ROSA26, provides predictable and stable integration sites that minimize disruption of endogenous genes, reduce the risk of insertional mutagenesis, and limit epigenetic silencing (Aznauryan et al., 2022; Oceguera-Yanez et al., 2016; Vlassis et al., 2023). Incorporating safe harbor targeting in iPSC-derived immune cells may ensure consistent transgene and safety switch expression across clones, streamlines quality control, and enhances reproducibility for clinical-scale manufacturing.

Rigorous quality control measures, clone screening, and advanced genome surveillance tools, including long-read sequencing and single-cell multi-omics, are essential to ensure the safety, fidelity, and reproducibility of iPSC-derived cell products intended for clinical use (Kuroda et al., 2015; Yasuda et al., 2024). iNKs offer a distinct advantage over cord blood- or PBMC–derived NK cells in quality control, as their clonal and renewable nature enables systematic screening and consistent characterization. However, rigorous monitoring is required throughout their manufacturing to limit the inevitable genetic drifting occurring with massive cell expansion (Merkle et al., 2017). Moreover, the potential risk of malignant transformation due to residual undifferentiated iPSCs must be carefully mitigated through effective purification strategies and sensitive detection assays prior to clinical application. However, the risk of residual undifferentiated iPSC may be less dangerous than originally anticipated with the recent demonstration that these cells are recognized and rejected by NK cells in humanized mice (Benabdallah et al., 2019). Nevertheless, incorporation of fail-safe mechanisms, such as inducible suicide genes (e.g., iCaspase-9 or Herpes Simplex Virus 1-thymidine kinase), provides an added layer of control to eliminate aberrant or unwanted cells post-infusion if necessary. For example, the iC9/Chemical Inducer of Dimerization (CID) system enables the selective activation of apoptosis in modified cells upon administration of a chemical inducer of dimerization, enhancing the safety profile of iPSC-derived therapeutic cell products (Ando et al., 2015).

### From developmental immaturity to therapeutic function in iNK cells

A major obstacle in the clinical translation of iPSC-based immunotherapies is the generation of immune effector cells that faithfully recapitulate the phenotypic and functional properties of their adult somatic counterparts. Although current differentiation strategies have made substantial advances (Budagova et al., 2025; Goldenson et al., 2022; Karagiannis and Kim, 2021), iPSC-derived immune cells, including iNK cells, often retain developmental features distinct from mature peripheral NK cells. These differences can influence cytokine secretion, cytotoxicity, persistence, and responsiveness in therapeutic settings (Huyghe et al., 2024; Nianias and Themeli, 2019). Such discrepancies arise from both the divergence of *in vitro* differentiation from native hematopoietic niches and the intrinsic developmental plasticity of iPSCs. An ongoing challenge in the field of iPSC-based immunotherapy is the refinement of iPSC differentiation protocols to generate immune cells that faithfully recapitulate the phenotypic and functional attributes of their adult somatic counterparts (Netsrithong et al., 2024; Studer et al., 2015; Xu et al., 2023).

Continued efforts to fine-tune differentiation trajectories will be essential to further align iNK cells with adult-like functionality while leveraging their unique developmental properties to enhance therapeutic efficacy. Producing iNK cells from iPSCs is a stepwise process in which iPSCs are first directed into mesodermal and then hematopoietic progenitors before becoming common lymphoid progenitors. Under defined cytokine and microenvironmental conditions, these lymphoid progenitors then mature into iNK (or iT) cells, and refining each stage is key to achieving more adult-like functionality and greater therapeutic efficacy (reviewed in Budagova et al., 2025). In brief, both stromal cell–based (2D) and embryoid body (EB)–based (3D) differentiation systems for the derivation of iNK cells have been well established, typically employing sequential cytokine cocktails to guide lineage specification. For example, the Kaufman group’s original two-step protocol generated lymphoid progenitors within 11 days in EB cultures supplemented with SCF, BMP4 and VEGF on mouse embryonic fibroblast (MEF) feeders, followed by 4–5 weeks of differentiation in IL-3, IL-15, SCF and FLT3L (Hermanson et al., 2015). Expansion was achieved on K562 feeder cells expressing membrane-bound IL-21 (Zhu and Kaufman, 2019). Later refinements replaced feeder cells with defined, xeno-free media and porcine gelatin substrates to improve reproducibility and clinical compatibility. Other groups have introduced sequential cytokine exposures or high-throughput 3D spheroid platforms (e.g. AggreWell microwells) to generate large numbers of uniform EBs (Euchner et al., 2021). Recently, fully defined, feeder-free culture systems have enabled large-scale production of iPSCs and their differentiation into iNK cells with potent anti-tumor cytotoxicity (Qin et al., 2024; Schmit et al., 2024).

iNK cells derived from optimized differentiation protocols often exhibit a highly activated phenotype, marked by elevated expression of NKG2D and the natural cytotoxicity receptors NKp30, NKp44, and NKp46. These cells also show increased levels of effector molecules, including perforin, granzyme B, and TRAIL, along with enhanced secretion of cytokines such as IFN-γ and TNF-α (Huyghe et al., 2024; Li et al., 2018; Thongsin et al., 2024). This phenotypic profile correlates with enhanced ADCC, heightened direct tumor cell lysis, and improved responsiveness to activating cytokines, collectively underpinning the strong antitumor activity of iNK cells that makes them phenotypically resemble highly active primary NK cells (Cichocki et al., 2020). An emerging strategy to exploit the natural cytotoxicity receptor repertoire of NK cells and enhance ADCC-like antitumor activity involves combining NK cell therapies with bispecific NK cell engagers (BIKEs). These fusion proteins that combine antibody-derived domains targeting NK-receptors (such as NKp46, NKp30, or CD16), with tumour targeting domains (Nikkhoi et al., 2024); BIKEs are analogous to more clinically advanced T-cell engager therapies. Combinatorial BIKE + NK approach has shown promise clinically using allogeneic primary NK cells (Nieto et al., 2025), and could offer a compelling route to flexible retargeting and functional enhancement of iNK cell therapies (Chiu et al., 2021).

Despite their elevated expression of activation markers and high ADCC phenotype, iNK cells seem to retain a slightly more developmental and immature transcriptional signature (e.g., fewer KIR receptors, low CD57 expression, and higher proportion of CD56^bright^ population) (Euchner et al., 2021; Kumar et al., 2025; Thongsin et al., 2024; Zeng et al., 2017; Zhang et al., 2024). iNK cells can also vary in CD16 expression compared to PB-NK cells (Goldenson et al., 2022; Knorr et al., 2013; Kumar et al., 2025; Li et al., 2018; Meng et al., 2023; Thongsin et al., 2024; Woll et al., 2005). This variability has been attributed, in part, to differences between donor and iPSC clones rather than the differentiation protocol (Knorr et al., 2013), highlighting an advantage of pre-screening iPSC lines that generate iNK cells with high CD16 expression (Huang et al., 2022). Notably, as the field has matured, genetic engineering of CD16, most commonly through the introduction of high-affinity, non-cleavable variants, has now become a standard approach to overcome endogenous variability, stabilize surface expression, and enhance ADCC, thereby reducing batch-to-batch and donor-related inconsistencies and enabling more uniform off-the-shelf iNK cell products. Interestingly, comparative studies have also shown that both feeder-based and feeder-free protocols can generate functional iNK cells, but they yield phenotypically and transcriptionally distinct populations. Huyghe *et al* compared two iNK cell differentiation protocols using the same iPSC line: a short-term, feeder-free method that recapitulates primitive hematopoiesis, and a lymphoid feeder–based method that models definitive hematopoietic differentiation (Huyghe et al., 2024). Both approaches successfully generated functional iNK cells. However, iNK cells derived from the lymphoid-based protocol exhibited a more mature, activated profile with elevated cytotoxic receptor expression and stronger antitumor activity against cancer cell lines. In contrast, iNK cells produced under feeder-free conditions, though attractive for clinical translation, displayed a less mature phenotype with reduced functional potency (Huyghe et al., 2024). These findings underscore that the differentiation pathway influences iNK cell identity and functionality, emphasizing the need to tailor protocols to the intended clinical application in adoptive immunotherapy.

Beyond refining cytokine combinations and culture formats, a key strategy to address the maturity limitations involves recreating critical spatial, mechanical, and biochemical niche cues that guide immune cell development *in vivo*. Next-generation differentiation approaches are increasingly leveraging tissue-specific niche components, such as stromal co-culture systems, decellularized extracellular matrices and organoid-based thymic scaffolds to recapitulate the temporally regulated environments of the bone marrow, thymus, and secondary lymphoid tissues (Cerneckis et al., 2024; Iriguchi et al., 2021; Montel-Hagen et al., 2019; Seet et al., 2017; Trotman-Grant et al., 2021; Wei et al., 2025). These engineered niches provide the instructive signals necessary for guiding lineage commitment, functional maturation, and antigen responsiveness. However, although iNK cells may not fully mirror the phenotype or functionality of mature adult peripheral counterparts, focusing exclusively on refining differentiation strategies may miss the bigger opportunity. iNK cells already display potent effector properties and expression of native cytotoxicity receptors. Thus, rather than exclusively striving for complete phenotypic convergence, an emerging paradigm is to engineer “function-first” iNK cells endowed with enhanced persistence, exhaustion resistance, optimized trafficking, and bespoke receptor repertoires. This combined developmental and engineering perspective reframes iPSC differentiation not merely as a means to mimic adult NK cells, but as a platform to create next-generation, clinically optimized cytotoxic iNK cells.

## Future-proofing iPSC-derived immunotherapies

### Engineering and manufacturing foundations for scalable iPSC immunotherapies

A major translational challenge in the development of iPSC-derived immune cell therapies lies in both the variability of differentiation outcomes and the ability to scale production for clinical and commercial use. This challenge is compounded by the well-recognized diversity that exists not only between distinct iPSC lines, but also among clones derived from the same parental line, reflecting donor-specific genomic features and residual epigenetic influences inherited from the somatic cell of origin (Kyttälä et al., 2016; Nath et al., 2023). As a result, the starting iPSC material can strongly influence differentiation efficiency, functional maturity, and manufacturing reproducibility. A key advantage of clonal iPSC selection is that it enables the identification of lines with favorable properties for genome engineering, stable expansion, and efficient differentiation into the desired immune lineage, thereby improving manufacturing consistency and product standardization (Nath et al., 2023). Nevertheless, this line- and clone-dependent variability necessitates systematic screening to identify candidates with superior yield, differentiation potential, and functional performance before large-scale manufacturing. Accordingly, establishing rigorous quality benchmarks, such as genomic integrity, epigenetic state, transcriptional signatures, and lineage competence, is essential to ensure reproducibility across laboratories and consistency in manufacturing workflows (Barry et al., 2026; Hägg et al., 2026; Madrid et al., 2024; Pantazis et al., 2022; Turner, 2021). Such variability also carries important downstream implications for process standardization, release criteria, lot-to-lot comparability, and regulatory assessment of product consistency.

These clonal considerations are further complicated by CAR design, as the architecture of the CAR construct, including the antigen-binding domain, hinge and transmembrane regions, intracellular signaling domains, and expression level, can influence not only target killing, but also lineage differentiation trajectories, proliferation, persistence, exhaustion, trafficking, and *in vivo* half-life (Li et al., 2023; Li et al., 2018; Sterner and Sterner, 2021; Yoo et al., 2024). Thus, clone selection and CAR engineering should be viewed as interdependent design parameters that jointly influence the phenotype, functionality, and manufacturability of the final cell product. In parallel, scaling manufacturing processes from research-grade to clinical-grade production has been slower and more difficult than expected for those developing therapies in this space, and still presents significant logistical and technical hurdles today. Robust, reproducible, and cost-effective bioprocessing platforms are needed to support the differentiation, expansion, and cryopreservation of engineered iNK cells under Good Manufacturing Practice (GMP) conditions. Transitioning to industrial-scale production requires the adoption of closed-system bioreactors, automation technologies, and real-time monitoring systems, along with standardized quality control and release assays to assess cell identity, purity, potency, and function (Nogueira et al., 2020). Together, overcoming variability in iPSC quality and stability and addressing the complexities of large-scale manufacturing represent critical steps toward realizing the full potential of off-the-shelf iPSC-derived immunotherapies.

### Regulatory and commercial pathways for the clinical translation of iPSC immunotherapies

Furthermore, regulatory pathways for gene-edited, off-the-shelf iPSC-based immune therapies remain complex and are still evolving (Song et al., 2024). Harmonizing regulatory frameworks, collaborative forums, early engagement with regulators, and clearer guidance on product characterization, quality control, safety, potency, and long-term monitoring will be essential to streamline approvals and support broader international adoption (Madrid et al., 2024; Song et al., 2024). At the same time, successful commercial translation will require scalable manufacturing enabled by modular platforms, automation, clear process and release criteria, and stronger public–private partnerships to address financial and regulatory barriers (Madrid et al., 2024). Although access to GMP-grade, clinical-quality iPSC lines and haplobanks (Escribá et al., 2024) is improving, their commercial availability remains inconsistent, with continued limitations related to cost, restricted access, variable documentation standards, donor consent provisions, and differing institutional or jurisdictional requirements. Important challenges also remain around standardizing donor consent, raw material qualification, and commercial licensing terms across jurisdictions. Licensing is especially complex in this space because freedom-to-operate may depend on access to multiple enabling technologies, including iPSC reprogramming platforms, gene-editing and vector technologies, proprietary CAR designs and manufacturing processes, creating a complex intellectual property landscape that can pose major barriers to translation and commercialization. Ultimately, progress will depend on a more integrated translational ecosystem that connects research, industrial development, and healthcare adoption through coordinated funding, strategic partnerships, improved regulatory guidance, and cross-sector collaboration.

It is worth noting that to date, the vast majority of iNK cell clinical studies have reported low toxicity, lack of treatment related patient deaths, and no reports of cell-product associated malignancies (Table 2). In the future, as our understanding and control improves for iNK cell therapies, iPSCs could become a safe cellular chassis for more complex cell therapy approaches beyond cancer and cytolytic applications. In parallel, ethical and equity considerations remain to be addressed, particularly around equitable access across genetic, geographies, and economically diverse jurisdictions, to prevent widening health disparities as these advanced therapeutic technologies mature. Looking ahead, the field is poised for significant transformation as programmable iPSC platforms continue to evolve (Xiong and Zheng, 2024). Advances in synthetic biology, non-viral gene editing, delivery technologies, and machine learning-driven design are enabling the creation of highly modular and adaptable cell therapies. These next-generation systems offer the ability to rapidly reconfigure immune cells with novel targeting moieties, inducible safety switches, microenvironment-specific resistance mechanisms and logic gates tailored to unique disease contexts. As such, iPSC-derived immune therapies are emerging not only as powerful tools for cancer and personalized therapies, but also as a cornerstone of future precision medicine where engineered cell therapies can be customized, manufactured at scale, and deployed broadly with precision, safety, and speed.

## Conclusion

The convergence of iPSC technology and advanced genome engineering is driving a paradigm shift toward off-the-shelf, programmable immunotherapies poised to overcome the limitations of current cell-based treatments. As these platforms continue to evolve, they promise to deliver cellular medicines that are not only safe and effective, but also scalable, customizable, and more accessible. By enabling the creation of standardized, yet highly customizable immune cell therapies from a single clonal source, iPSC-derived immunotherapies are reshaping the very definition of personalized medicine from treatments tailored to individual patients, to off-the-shelf platforms designed to deliver precision therapies at scale. Looking ahead, these therapies have the potential to overcome the logistical, economic, and supply-chain limitations of autologous cell therapies. These innovations hold promise not only for oncology but also for autoimmune diseases and chronic infections ultimately redefining precision medicine.
